# Processus organisationnels et pratiques cliniques pour la gestion de la douleur chronique en soins primaires : une offre de service découlant du plan d’action en douleur chronique 2021-2026 ministériel du Québec, Canada

**DOI:** 10.1080/24740527.2026.2672488

**Published:** 2026-07-09

**Authors:** G. Lavoie-Dias, T. Augière, Y. Couturier, N. -K. Jérôme, M. Choinière, G. Arsenault-Lapierre, H. Beaudry, L. Beauregard, H. Bergman, A. Boulanger, D. Chaussé, J. Côté, C. Hudon, L. Guénette, A. Lacasse, J. Laliberté, M. O. Martel, N. Mercier, A. M. Pinard, K. Rice, Y. Tousignant-Laflamme, N. Tremblay, I. Vedel, R. Visca, T. H. Wideman, M. G. Pagé

**Affiliations:** a Département de psychologie, Faculté des arts et science, Université de Montréal, Montréal, QC, Canada; b Centre de recherche du Centre hospitalier de l’Université de Montréal (CRCHUM), Montréal, QC, Canada; c École de travail social, Faculté des lettres et sciences humaines, Université de Sherbrooke, Sherbrooke, QC, Canada; d Département d’anesthésiologie et médecine de la douleur, Faculté de médecine, Université de Montréal, Montréal, QC, Canada; e Centre de recherche et d’expertise en gérontologie sociale, CIUSSS COMTL, Montréal, QC, Canada; f Department of Family Medicine, Faculty of Medicine and Health Sciences, McGill University, Montreal, QC, Canada; g Quebec Pain Research Network (QPRN), Sherbrooke, QC, Canada; h Ministère de la Santé et des services sociaux, Québec, QC, Canada; i Centre de gestion de la douleur, Centre hospitalier de l’Université de Montréal (CHUM), Montréal, QC, Canada; j Patient-partenaire, QC, Canada; k Faculté des sciences infirmières, Université de Montréal, Montréal, QC, Canada; l Département de médecine de famille et médecine d’urgence, Faculté de médecine et des sciences de la santé, Université de Sherbrooke, Sherbrooke, QC, Canada; m Faculté de Pharmacie, Université Laval, Québec, QC, Canada; n Département des sciences de la santé, Université du Québec en Abitibi-Témiscamingue, Rouyn-Noranda, QC, Canada; o Faculty of Dentistry, McGill University, Montreal, QC, Canada; p CHU de Québec-Université Laval, Québec, QC, Canada; q Faculté de médecine, Université Laval, Québec, QC, Canada; r École de réadaptation, Faculté de médecine et des sciences de la santé, Université de Sherbrooke, Sherbrooke, QC, Canada; s Réseau universitaire intégré de santé et de services sociaux de l’Université de Montréal, Montréal, QC, Canada; t Réseau universitaire intégré de santé et de services sociaux de l’Université McGill, Montréal, QC, Canada; u School of Physical and Occupational Therapy, Faculty of Medicine and Health Sciences, McGill University, Montreal, QC, Canada

**Keywords:** Douleur chronique, implantation, services interprofessionnels, méthodes mixtes, processus organisationnels, soins primaires

## Abstract

**Introduction:**

La douleur chronique demeure un enjeu majeur, particulièrement en soins primaires où l’accès à des services appropriés et coordonnés est limité. Pour y répondre, le Plan d’action ministériel québécois en douleur chronique 2021–2026 vise à améliorer l’accès à des soins intégrés grâce à de nouveaux projets de services interprofessionnels en soins primaires.

**Objectif:**

Documenter la mise en œuvre de ces projets en analysant les processus décisionnels, organisationnels et cliniques, ainsi que les innovations et défis observés.

**Méthodes:**

Une étude de cas multiméthode a été menée dans cinq projets. Dix gestionnaires et responsables ont participé à des entretiens semidirigés et rempli une enquête organisationnelle. Les données qualitatives et quantitatives ont été analysées pour identifier les facilitateurs et les obstacles à l’implantation.

**Résultats:**

Tous les projets ont implanté des modèles interprofessionnels, mais avec une hétérogénéité importante selon les ressources disponibles. Le triage est principalement assuré par les infirmières, avec une implication médicale limitée. Des pratiques collaboratives dynamiques et qui renforcent la continuité des soins et services ont émergé. Malgré tout, les équipes font face à des défis organisationnels majeurs, dont le financement temporaire, la pénurie de personnel et une coordination intersectorielle fragile.

**Conclusion:**

Ces services représentent une avancée significative, mais leur pérennité requiert un soutien institutionnel et un financement durable.

## Résumé

**Introduction:** Chronic pain remains a major issue, particularly in primary care, where access to appropriate, coordinated services is limited. To address this issue, Quebec’s 2021–2026 Ministerial Action Plan for Chronic Pain aims to improve access to integrated care through new interprofessional primary care service projects.

**Objective:** To document the implementation of these projects by analyzing decision-making, organizational, and clinical processes, as well as observed innovations and challenges.

**Methods:** A multi-method case study was conducted across five projects. Ten managers and project leads participated in semi-structured interviews and completed an organizational survey. Qualitative and quantitative data were analyzed to identify facilitators and barriers to implementation.

**Results:** All projects implemented interprofessional models, but with considerable heterogeneity depending on available resources. Triage was mainly performed by nurses, with limited physician involvement. Collaborative practice dynamics emerged, helping to strengthen continuity of care and services. Nevertheless, teams faced major organizational challenges, including temporary funding, staffing shortages, and fragile intersectoral coordination.

**Conclusion:** These services represent a significant step forward, but their sustainability requires institutional support and stable funding.

## Introduction

On estime qu’environ une personne sur cinq au Canada^1^ vit avec de la douleur chronique (douleur qui persiste > 3 mois),^2^ laquelle découle de l’interaction complexe de divers facteurs biopsychosociaux.^3^ En effet, l’expérience de la douleur n’est pas directement proportionnelle à l’étendue de la blessure et peut exister en l’absence de lésion; la douleur représente plutôt le produit de facteurs biologiques (par ex. nociception, gênes, le sexe, l’âge), psychologiques (par ex. émotions telles que la peur ou bien l’anxiété ou la dépression, cognitions telles que les croyances sur la douleur, le stress, et les capacités d’adaptation) et sociaux (par ex. éléments culturels, support social, vulnérabilité économique). La douleur chronique est une condition hétérogène qui englobe les douleurs chroniques primaires (une douleur persistante pouvant toucher une ou plusieurs régions anatomiques, qui est associée à de la détresse émotionnelle et à des limitations fonctionnelles et qui n’est pas mieux expliquée par une autre maladie ou un autre diagnostic) pour lesquelles la douleur est considérée comme une affection à part entière et les douleurs secondaires (une douleur persistante attribuable à une maladie ou à une lésion identifiable où la douleur est considérée comme un symptôme d’une affection sousjacente (par ex. maladie inflammatoire).^2^ Les approches interprofessionnelles qui regroupent différents professionnels de la santé (par ex. médecin, physiothérapeute, psychologue, ergothérapeute) offrant une prise en charge globale de la douleur chronique sont reconnues comme étant le modèle de soins idéal (*gold standard*) pour les douleurs chroniques^4,5^ et ont démontré leur efficacité malgré une hétérogénéité des modèles existants et le besoin de données probantes de haute qualité.^6^ En contraste aux approches multidisciplinaires qui se caractérisent par l’intervention en parallèle de différents types de professionnels de la santé provenant de disciplines diverses qui poursuivent chacun des objectifs en lien avec leur expertise, les approches interprofessionnelles exigent la collaboration entre les professionnels, un processus de décisions partagées pour atteindre un objectif commun et une coresponsabilité des soins avec les personnes malades.^7^ Un manque flagrant de ressources, une fragmentation des soins et une formation insuffisante du personnel soignant à l’égard des spécificités de la douleur chronique font en sorte que les soins pour les personnes qui en sont atteintes ne sont pas optimaux.^8^

L’enjeu de politique publique lié à la prise en charge de la douleur chronique au Québec (Canada) a été identifié il y a plusieurs années. Déjà en 2006, l’Agence d’évaluation des technologies et des modes d’intervention en santé du Québec a proposé la création de centres d’expertise en gestion de la douleur chronique afin de déployer des services spécialisés sur l’ensemble du territoire, d’améliorer la formation des cliniciens et de développer la recherche sur la douleur.^9^ En 2019, le Groupe de travail sur la douleur de Santé Canada identifiait le besoin de renforcer les capacités de gestion de la douleur chronique et l’accès à des soins opportuns, équitables et axés sur le patient.^1^

Dans la foulée de ces initiatives et afin de répondre aux besoins criants de cette clientèle, le ministère de la Santé et des Services sociaux du Québec a élaboré en 2021 un Plan d’action pour la gestion de la douleur chronique contenant une série de mesures visant une meilleure accessibilité à des services de qualité, un soutien accru aux médecins de famille par des équipes interprofessionnelles pour les situations complexes et une meilleure coordination des services.^10^ Les grandes orientations de ce Plan d’action incluent la prise de décision partagée avec les patientes et les patients, l’amélioration des trajectoires de services et l’offre de soins basés sur les bonnes pratiques cliniques et les données probantes. Les principes directeurs de ce Plan d’action sont formulés autour de cinq axes principaux, notamment l’accessibilité, le rôle de la patiente et du patient, le transfert des connaissances, l’évaluation et l’amélioration de la qualité de même que la gouvernance.

La mesure phare de ce Plan d’action est la constitution d’équipes interprofessionnelles en soins primaires dédiées à la prise en charge de la douleur chronique. Afin d’opérationnaliser cette mesure, un appel à projets a été lancé en 2023 par le ministère pour développer localement six offres de services interprofessionnels en soins primaires, avec l’intention de déployer éventuellement ce modèle de soins à l’échelle de la province. Cet appel à projet avait plusieurs objectifs, dont (1) l’établissement d’une offre de services interprofessionnels pour la gestion de la douleur chronique dans les services de proximité, (2) le développement des trajectoires d’accès aux plateaux diagnostics et aux interventions techniques, (3) la formation et le soutien des professionnels de la santé et des services sociaux en gestion de la douleur, (4) la mise en place de la fonction de gestion de cas en douleur chronique intégrant et coordonnant les interventions de l’équipe intra et interétablissement, (5) l’amélioration de l’évaluation de la patientèle pour établir adéquatement la prise en charge selon les valeurs et les besoins des individus et (6) l’autonomie et la prise de décision partagée entre la patientèle et l’équipe interprofessionnelle. La patientèle qui devait être visée par les services interprofessionnels était les adultes et les personnes âgées vivant avec de la douleur subaiguë ou chronique en excluant les soins de fin de vie. La prise en charge de la patientèle serait localisée dans les services de proximité par un médecin et les membres d’une équipe interprofessionnelle. Pour soutenir la réussite et la mise en œuvre de ces services interprofessionnels, il était important de procéder à une évaluation rigoureuse des composantes de leur implantation et de leurs impacts.

Des offres de services interprofessionnels en soins primaires existent déjà ailleurs ce qui porte à croire qu’il est possible de créer et de pérenniser de tels modèles. Le *Veterans Health Administration* (VHA), l’un des systèmes intégrés ayant déployé durablement des cliniques interprofessionnelles en soins primaires,^11^ présente plusieurs similitudes avec les projets déployés dans le cadre du Plan d’action du Québec: la colocalisation des professionnels, l’adoption d’un modèle biopsychosocial, la collaboration entre professionnels et la prise en charge multimodale. D’autres travaux soulignent que l’interprofessionnalité en soins primaires améliore les résultats cliniques^5^ et favorise l’intégration de traitements non pharmacologiques.^12^ Par ailleurs en France, 243 structures de douleur chronique existent pour un total de 69 millions d’habitants et un territoire qui est le tiers de celui du Québec. Malgré leur implantation, l’accès aux soins demeurent un enjeu important.^13^ En Australie, plusieurs programmes de prévention secondaire de la douleur chronique existent dans le réseau de soins primaires, mais les données probantes pour démontrer leur efficacité, leur coût-efficacité et leur processus d’implémentation sont insuffisantes.^14^ Les particularités des écosystèmes de santé locaux font en sorte qu’un modèle peut être implanté avec succès dans un système de soins, mais peut échouer dans un autre, d’où l’importance de documenter rigoureusement les processus de mise en œuvre d’innovations cliniques.

La présente étude visait donc à documenter les processus décisionnels, organisationnels et cliniques mis en place dans ces projets locaux ayant appliqué pour obtenir un financement afin d’analyser la concordance de leur offre de services avec les axes prioritaires du Plan d’action et d’identifier les innovations locales porteuses de valeurs du point de vue des gestionnaires et responsables des projets. Ces projets sont déployés au Québec qui est une province canadienne avec un système de soins caractérisé par une forte intégration institutionnelle des services de santé au sein de centres intégrés de santé et de services sociaux, et donc plusieurs services de soins primaires y sont imbriqués. Ceci pourrait faciliter d’un point de vue théorique l’interprofessionnalité (par ex. ressources partagées, corridor de services), mais peut en complexifier la gouvernance et l’agilité organisationnelle. La douleur chronique se situe elle-même à l’interface des soins primaires (premier point de contact qui assure un suivi longitudinal) et des services spécialisés (cliniques de douleur) ce qui peut entraîner une redéfinition des rôles professionnels avec le déploiement de cliniques interprofessionnelles dans les services primaires. Finalement, la forte dépendance aux modèles de financement et à la rémunération médicale à l’acte crée un contexte particulier dans lequel des initiatives telles que les services interprofessionnels doivent s’adapter pour se déployer. Une étude rigoureuse visant à comprendre comment et sous quelles conditions ces services interprofessionnels se déploient permettra de générer des connaissances qui informeront non seulement la mise à l’échelle de ces projets le cas échéant, ainsi que la mise en œuvre de services similaires visant d’autres clientèles en santé.

## Méthodologie

### Devis

**Figure 1.**
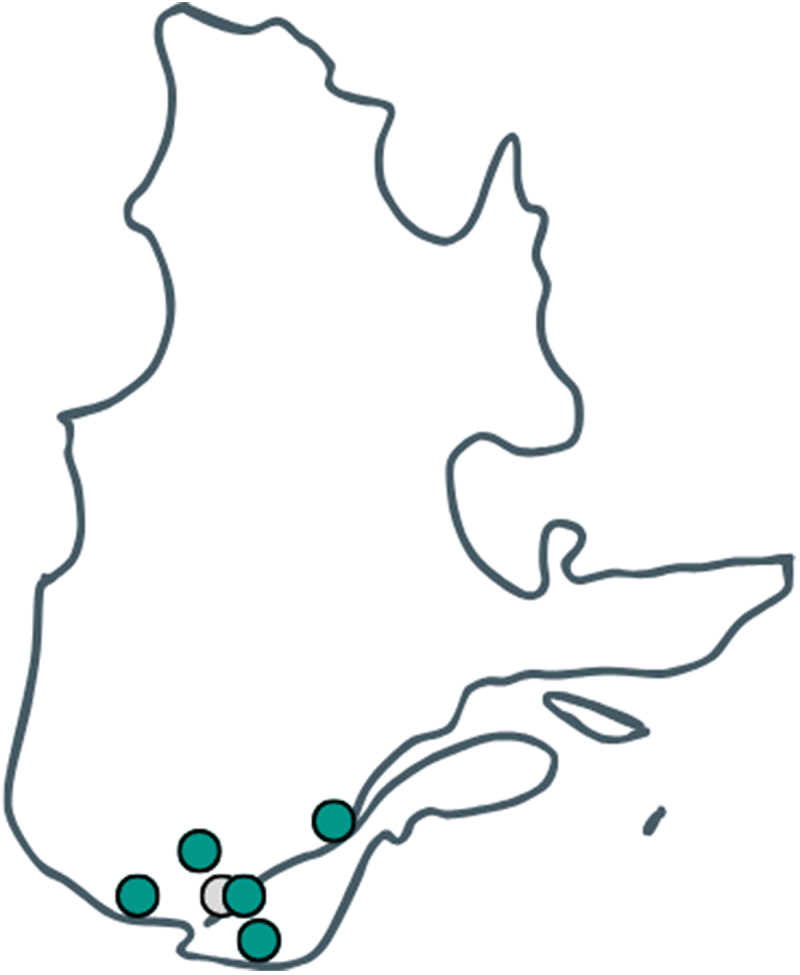
La Figure 1 illustre l’emplacement des six services interprofessionnels qui ont été mis sur pied. Les services qui sont inclus dans ce projet de recherche sont identifiés en vert alors que celui n’en faisant pas partie est en gris.

Cette étude de cas multiméthode^15^ visait à colliger et à trianguler des données qualitatives et quantitatives sur l’implantation de nouveaux projets de services interprofessionnels en gestion de la douleur chronique au Québec. Six projets locaux ont été déployés (voir Figure 1 pour leur emplacement géographique) et les résultats portent sur cinq d’entre eux; les données du sixième n’ayant pu être obtenues à l’intérieur des six premiers mois d’opération dû à des délais administratifs. Les résultats qualitatifs de cette étude au cœur du présent article, respectent les directives COREQ (*Consolidated criteria for reporting qualitative research*^16^) afin de garantir la transparence et la rigueur de la recherche qualitative (voir Annexe 1). Le projet a été approuvé par le comité d’éthique de la recherche du Centre hospitalier de l’Université de Montréal (CHUM) (MP02202411739).

### Posture épistémologique

Cette étude s’inscrit dans une posture épistémologique principalement postpositiviste reconnaissant qu’une réalité objective existe et peut être étudiée, mais n’est pas mesurable objectivement car l’étude de cette réalité est nécessairement influencée par les valeurs, les croyances et les biais présents dans le processus scientifique.^17^ Cette posture a guidé les choix méthodologiques de l’étude, notamment le recours à des méthodes qualitatives permettant d’explorer les expériences et les interprétations des participants, tout en intégrant une démarche réflexive quant à la production et à l’interprétation des données. Cette posture se traduit par une attention portée à la rigueur méthodologique, à la transparence des procédures et à la réflexivité tout au long du processus de recherche, tout en reconnaissant que les expériences vécues par les gestionnaires sont construites à l’interface de dimensions individuelles, contextuelles et organisationnelles.

L’équipe de recherche était composée de personnes chercheures, de personnes cliniciennes, de décideurs, de gestionnaires et de personnes patientes partenaires. Plusieurs personnes ont un vécu expérientiel en lien avec la douleur chronique et l’utilisation des soins de santé. Tous les membres ont été impliqués dans l’élaboration des questions de recherche, les choix méthodologiques, ainsi que l’interprétation des résultats.

### Recrutement et participantes

Toutes les gestionnaires principales et les responsables locales des projets ont été sollicitées par courriel dans les six mois suivant le début des activités cliniques, avec un taux de recrutement de 100%. Le consentement éclairé des participantes a été obtenu électroniquement via REDCap.

### Procédures

Chaque personne participante a pris part à un entretien individuel semi-dirigé en ligne mené par un ou deux membres de l’équipe de recherche (YC, ED, TA ou GLD) sur la plateforme Microsoft TEAMS. Les entretiens portaient sur l’offre de services, l’intégration des services dans la trajectoire de soins au sein de l’autorité régionale de santé à laquelle ils sont rattachés et les défis de mise en oeuvre. Les transcriptions automatiques ont été vérifiées et corrigées manuellement, sans retour vers les participantes pour vérification. La durée des entretiens variait entre 17 minutes et 44 minutes (médiane de 40 minutes avec un écart interquartile de 7 minutes). Les gestionnaires principales des équipes ont également répondu électroniquement à un questionnaire organisationnel structuré via REDCap, élaboré selon le cadre théorique consolidé de la science d’implantation pour la recherche (CFIR^18^; processus d’implantation, innovation, contexte interne et externe et caractéristiques des individus impliqués) et présenté selon les axes du Plan d’action et qui consistait en une série de questions à choix multiple ou unique et qui est disponible à l’Annexe 2.

### Plan d’action

Les données colligées ont été examinées en fonction des cinq axes du Plan d’action en douleur chronique.

#### Axe 1. Accessibilité

Ce premier axe a un double objectif, notamment d’améliorer la prise en charge de la douleur chronique et de faciliter l’accès à des soins et des services. Ces services visent à améliorer l’évaluation du patient et de la patiente, à développer une offre de services interprofessionnels et à coordonner la gestion des cas au travers du continuum de soins. Cet axe comporte deux sous-objectifs, notamment de favoriser l’interprofessionnalité et l’approche biopsychosociale pour améliorer la prise en charge, ainsi que l’amélioration des mécanismes d’accès aux services interprofessionnels.

#### Axe 2. Rôle de la patiente et du patient

Le deuxième axe vise à favoriser l’autonomie et la prise de décision répartie entre la patiente ou le patient et la professionnelle ou le professionnel de la santé afin de prodiguer des soins orientés sur les besoins des patientes et des patients et d’améliorer leurs connaissances.

#### Axe 3. Transfert des connaissances

Ce troisième axe reconnaît le besoin de soutenir, par des activités de formation continue, de services de mentorat et des consultations, les cliniciens et les cliniciennes dans le travail interdisciplinaire en gestion de la douleur chronique, en s’appuyant sur des données probantes. Ceci peut prendre la forme par exemple de la promotion de pratiques optimales, l’implantation d’un curriculum de compétences, l’évaluation de la qualité et l’optimisation de l’utilisation des résultats de recherche dans la pratique clinique.

#### Axe 4. Évaluation et amélioration de la qualité

Ce quatrième axe vise à déployer une culture d’amélioration continue et à encourager la pratique réflexive tant sur le plan clinique qu’organisationnel. Cet objectif met l’emphase sur la collecte de données médicoadministratives pour optimiser les trajectoires de soins et mesurer la variabilité des pratiques, permettre l’imputabilité et la reddition de comptes, et informer les prises de décision.

#### Axe 5. Gouvernance

Finalement, le dernier axe vise à assurer un suivi serré des initiatives déployées. Il favorise une définition claire des structures organisationnelles en place pour favoriser l’harmonisation des services en douleur chronique, et maintenir le financement de la recherche, des innovations et des coordonnateurs de réseau.

### Analyses

Les données qualitatives ont été analysées selon une approche descriptive principalement déductive^19^ à partir des axes du Plan d’action en douleur chronique, tandis que les données quantitatives sont présentées sous forme de statistiques descriptives. Une chercheuse (GLD) a généré un livre de code déductif en fonction des axes du Plan d’action qu’elle a ensuite appliqué aux données. Les données qualitatives ont été analysées manuellement sans l’aide d’un logiciel. Des rencontres fréquentes avec les autres membres impliqués directement dans les analyses (TA, YC, MGP) ont eu lieu afin de peaufiner les analyses et la compréhension des données. Les données qualitatives et quantitatives ont ensuite été intégrées, principalement en utilisant une approche narrative^20^ afin de dégager une compréhension contextualisée de l’implantation du Plan d’action au sein des cinq projets.

### Équipe de recherche

Les chercheurs directement impliqués dans la collecte des données et/ou des analyses (GLD, TA, YC, MGP) avaient une formation en recherche qualitative et provenaient des domaines de la psychologie, du travail social et de la réadaptation. Trois s’identifient comme des femmes et un comme un homme, avec des niveaux variés d’expérience (étudiante à la maîtrise à plus de 30 ans d’expérience en recherche qualitative). Une chercheuse est aussi clinicienne oeuvrant auprès de la patientèle vivant avec de la douleur chronique. Les chercheurs n’avaient pas de relation établie avec les participantes avant le début du projet de recherche, bien qu’ils aient été en étroite collaboration pendant celui-ci. Les qualifications académiques des chercheurs ont été partagées avec les participantes, ainsi que les objectifs du projet de recherche. Ces chercheurs n’avaient pas eux-mêmes une expérience vécue de douleur chronique, bien que des patientes et patients partenaires (JL, DC) font partie de l’équipe de recherche.

## Résultats

Les caractéristiques des dix participantes (taux de réponse de 100%; toutes étaient des femmes) sont présentées au Tableau 1. La majorité des gestionnaires avaient moins de cinq ans d’expérience (80%) et avaient une formation clinique (80%). Les réponses au questionnaire organisationnel sont présentées au Tableau 2.

**Tableau 1.** Caractéristiques des participantes’ with an e at participantes.

Caractéristiques des participants	N (%)
Occupation	
Gestionnaires	8 (80)
Cliniciennes	2 (20)
Proportion du travail consacré à cette occupation	
Tout son temps	5 (62,5)
Majorité de son temps	1 (12,5)
Minorité de son temps	2 (25)
Donnée manquante	2
Secteur desservi	
GMF	1 (10)
GMF-U	2 (20)
Cliniques spécialisées et surspécialisées	4 (40)
CLSC	1 (10)
Hôpital	1 (10)
CIUSSS	1 (10)
Nombre d’années en poste	
0–5 ans	7 (70)
6–10 ans	1 (10)
11–15 ans	1 (10)
16–20 ans	1 (10)
Nombre de personnes sous leur supervision	
0–25	2 (25)
26–50	3 (37,5)
51–75	3 (37,5)
Donnée manquante	2
Formation clinique	
Travail social	2 (20)
Physiothérapie	4 (40)
Médecin spécialiste	1 (10)
Autre	3 (30)
Âge (années)	
26–35	2 (20)
36–45	4 (40)
46–55	4 (40)
Genre	
Femme	10 (100)

GMF: Groupe de médecine familiale GMF-U: Groupe de médecine familiale universitaire CLSC: Centre local de services communautaires CIUSSS: Centre intégré universitaire de santé et de services sociaux.

**Tableau 2.** Résultats des enquêtes organisationnelles et des pratiques cliniques déployées dans les cinq services interprofessionnels.

	Domaine mesuré	N (%) cliniques ou médiane (écart interquartile)	
Axe accessibilité			
Objectif 1: Favoriser l’interprofessionnalité et l’approche biopsychosociale pour améliorer la prise en charge			
Composition des équipes	Professionnelles et professionnels embauchés dans les équipes Ergothérapeute Infirmière Kinésiologue Nutritionniste Pharmacien Physiothérapeute/Technologue en physiothérapie Psychologue Spécialiste d’activités cliniques Travailleur social	4 (80) 4 (80) 5 (100) 1 (20) 1 (20) 4 (80) 2 (40) 1 (20) 3 (60)	
Plan de soin	Utilisation d’un plan de soin interprofessionnel		
	Plan de soin formel	3 (60)	
	Plan de soin informel	2 (40)	
	Rédaction du plan de soin		
	Individuellement par chaque professionnel	3 (60)	
	Autre	2 (40)	
	Rôle du médecin dans l’élaboration du plan de soin		
	Médecin contribue	1 (20)	
	Médecin ne contribue pas	1 (20)	
	Autre	3 (60)	
Objectifs d’implantation	Priorités locales identifiées qui s’alignent avec les orientations du Plan d’action		
	Évaluation interprofessionnelle	5 (100)	
	Collaboration interprofessionnelle	4 (80)	
	Prise de décision partagée	3 (60)	
	Autonomie des patients, collaboration entre niveaux	2 (40)	
	Accès aux plateaux diagnostiques, interventions techniques, représentation des patients, formation	1 (20)	
Objectif 2: Amélioration des mécanismes d’accès			
Partenariat	Partenariat entre la 1^ière^ et la 2^ème^ ligne		
	Réunion multi-acteurs (GMF-U + clinique spécialisée)	2 (40)	
	Clinique spécialisée seule	1 (20)	
	Aucune réunion	1 (20)	
	Non-réponse	1 (20)	
	Disponibilité du soutien expert (/10)	8 (8–9)	
	Facilité de communication avec le référent (/10)	6,5 (4–8,5)	
Ciblage des patients et corridors de référencement	Stratégies de référencement adoptées		
	Desservies par GMF-U affiliés	5 (100)	
	En attente de 2e ligne	3 (60)	
	Sans médecin de famille	3 (60)	
	Besoins complexes	2 (40)	
	Objectif « augmenter le volume »	2 (40)	
	Cas urgents	2 (40)	
	Procédures d’accès		
	Référence à l’équipe (triage interne)	5 (100)	
	Liste d’attente spécialisée	2 (40)	
	GAP	1 (20)	
	Procédure en développement	1 (20)	
	Autre	1 (20)	
	Processus de priorisation		
	Professionnel de l’équipe	2 (40)	
	Demandeur GMF-U	1 (20)	
	Ordre d’arrivée	1 (20)	
	Autre	1 (20)	
	Élaboration des critères de priorisation		
	Équipe seule	2 (40)	
	Avec GMF-U	1 (20)	
	Autre	2 (40)	
Axe rôle de la patiente et du patient			
Autosoins	Développement d’outils et de stratégies au soutien de l’autogestion		
	Outils d’aide à la décision	2 (40)	
	Communauté de pratique	1 (20)	
	En développement	2 (40)	
	Aucun	1 (20)	
	Autre	1 (20)	
Patient partenaire	Inclusion d’un partenaire		
	Oui	4 (80)	
	Non	1 (20)	
	Rôle des patients partenaires		
	Centré sur les activités	3 (60)	
	Participation aux décisions stratégiques et à la planification clinique	1 (20)	
Axe transfert des connaissances			
Soutien expert	Disponibilité du soutien expert (/10)	8 (8–9)	
Conditions organisationnelles mises en place pour optimiser le transfert des connaissances	Perception du secteur d’activité comme étant un facilitateur ou un frein au transfert des connaissances	Facilitateurs	Freins
	Formation des professionnels en gestion de la douleur Formation des professionnels à la collaboration interprofessionnelle	4 (80) 2 (40)	1 (20) 0 (0)
Axe évaluation et amélioration de la qualité			
Conditions organisationnelles mises en place pour améliorer la qualité de la pratique	Perception du secteur d’activité comme étant un facilitateur ou un frein à l’amélioration de la qualité de la pratique	Facilitateurs	Freins
	Coordonner l’équipe avec les autres partenaires (GMF, clinique spécialisée, ministère)	1 (20)	1 (20)
	Coordination interne	2 (40)	1 (20)
	Définition de critères d’accès et de priorisation	2 (40)	0 (0)
	Développement d’outils cliniques	2 (40)	2 (40)
	Obligations de collecte d’indicateurs	0 (0)	3 (60)
Axe de gouvernance			
Conditions de gestion des services interprofessionnels	Perception du secteur d’activité comme étant un facilitateur ou un frein à la gestion des projets interprofessionnels	Facilitateurs	Freins
	Stratégie explicite du changement	3 (60)	0 (0)
	Définition des objectifs du projet	3 (60)	2 (40)
	Déterminer l’ampleur de l’offre du projet	2 (40)	2 (40)
	Gérer un projet avec un échéancier très court	1 (20)	2 (40)
	Durée du projet possiblement limité	1 (20)	3 (60)
	Opérationnalisation des ressources – disponibilité des espaces	1 (20)	3 (60)
	Charge de travail additionnelle pour les gestionnaires	1 (20)	3 (60)
	Difficulté de recrutement des cliniciens		
	Physiothérapeutes	3 (60)	
	Psychologues	2 (40)	
	Travailleurs sociaux	1 (20)	
	Autres professionnels	0 (0)	
	Pas un enjeu de recruter	0 (0)	

GMF-U: Groupe de médecine familiale universitaire.

**Figure 2.**
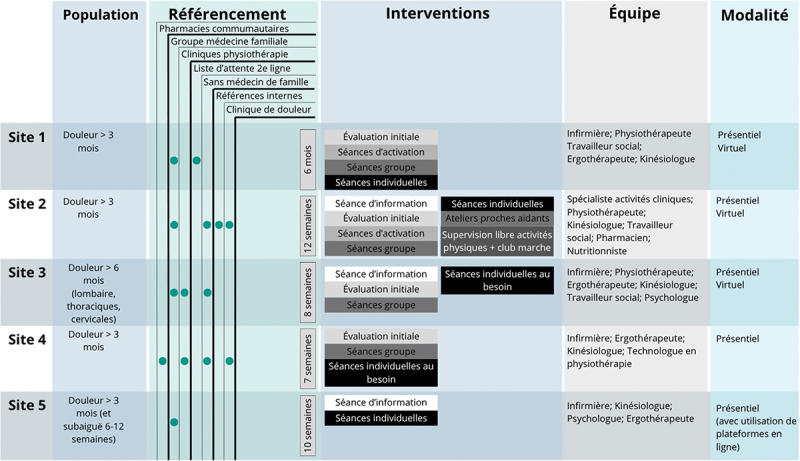
La figure présente cinq centres universitaires intégrés de santé et de services sociaux où se sont développés les différents projets pour la douleur chronique (horizontal). Les caractéristiques des populations ciblées, la source de référencement, les interventions offertes au sein des projets et la composition des équipes interprofessionnelles (vertical) sont également indiquées. Ces données ont été colligées principalement via le questionnaire d’enquête organisationnelle, ainsi que les entretiens avec les gestionnaires des projets.

Les caractéristiques des cinq projets sont présentées à la Figure 2. Tous les projets acceptaient des personnes qui vivaient avec une douleur depuis plus de trois ou six mois, et les sources de référencement étaient diverses, incluant les cliniques spécialisées, les références internes, les populations sans médecin de famille, les cliniques de physiothérapie, les pharmacies communautaires ou les Groupes de médecine familiale (GMF; regroupement de médecins et autres professionnelles et professionnels de la santé qui œuvrent en soins primaires au Québec) et tous les types de douleur chronique étaient admissibles. La durée du suivi offert variait entre deux et six mois. Tous les projets offraient une séance d’information ou une évaluation initiale, et les suivis offerts étaient composés de séances individuelles seulement, d’une combinaison de séances individuelles ou de groupe, ou bien principalement de séances de groupe. La composition des équipes variait aussi beaucoup d’un projet à un autre et pouvait inclure des kinésiologues, des physiothérapeutes, des infirmières, des travailleurs sociaux, des ergothérapeutes, des pharmaciens, des nutritionnistes, des psychologues et des spécialistes d’activités cliniques. Les soins qui y sont offerts sont sans frais pour la patientèle. Chaque projet avait un emplacement physique, soit dans des locaux sur le territoire desservi, ou bien à partir des locaux des Groupes de médecine familiale par exemple.

### Axe – Accessibilité

#### Objectif 1: Favoriser l’interprofessionnalité et l’approche biopsychosociale pour améliorer la prise en charge

Tel qu’attendu, les cinq projets ont déployé une offre de services interprofessionnels en soins primaires pour la population adulte. Comme indiqué à la Figure 2 et au Tableau 2, les modèles de soins développés sont hétérogènes, particulièrement quant à la composition des équipes, le rôle des médecins, les mécanismes de priorisation des patientes et des patients et les partenariats locaux. Malgré cela, l’interprofessionnalité constitue le pilier central des services offerts :

Le projet [nom du programme], c’est une équipe interdisciplinaire […] on amène des nouvelles approches en première ligne. (P1)

Les équipes ont été constituées de manière interprofessionnelle, bien que leur composition varie selon les projets. Les équipes regroupent entre cinq et huit professions (voir Figure 2), avec une présence systématique des kinésiologues (tous les projets), tandis que les psychologues (deux des cinq projets), les pharmaciens (un des cinq projets) et les nutritionnistes (un des cinq projets) sont moins représentés. Ceci s’explique en partie en raison d’une disponibilité limitée de ces disciplines :

On est chanceux de l’avoir [la psychologue] … c’est une ressource précieuse, mais (aurons nous) les moyens … en première ligne, d’avoir un psychologue comme ça partout? (P1)

Les contraintes organisationnelles en termes de dotation de postes, telles que le roulement, les surcroîts de tâche et les postes non permanents, accentuent également cette difficulté :

Il y a eu du roulement de personnel parce que c’est des surcroîts, ce ne sont pas des postes; comment avoir une rétention quand on n’a pas grand chose à offrir. (P2)

Ainsi, même si la vision idéale prévoyait une équipe complète incluant toutes les disciplines clés, la réalité de terrain a mené à une implantation partielle, davantage façonnée par les ressources disponibles.

Tous les projets ont adopté une stratégie d’élaboration de plan de soins interprofessionnel pour toutes les patientes et tous les patients (trois des cinq projets) ou pour un sous-groupe de patientes et de patients (deux des cinq projets): « c’est toujours de la intervention parce qu’on y croit. » (P2). Les discussions de cas soutiennent une compréhension globale de la patientèle: « l’objectif, c’est d’avoir une vue 360° du patient. » (P3).

Certains rôles professionnels s’élargissent, notamment celui du pharmacien intégré au volet psychosocial :

Le volet pharmaco est super important, mais en même temps, si on regarde au niveau de notre pharmacien, il y a un gros bout qui est le volet psychosocial aussi. […] Si ce n’est pas travaillé au niveau de l’hygiène de vie ou autre, il n’y aura pas d’incidence nécessairement. (P2)

Le rôle médical est, quant à lui, limité à une fonction de premier référent, parfois de répondant : « L’engagement du médecin est indirect […] on a un médecin répondant pour l’équipe. » (P4). En revanche, l’organisation des services repose beaucoup sur l’infirmière clinicienne, qui assume un rôle pivot dans la trajectoire des patientes et des patients « selon le niveau de complexité » (P4). La structure opérationnelle du système de santé rend cependant ce rôle complexe, car certaines tâches doivent obligatoirement être effectuées par un médecin : « S’il faut qu’il voie un psychiatre, la requête doit venir d’un médecin » (P5). Ceci crée parfois un écart entre la référence et la prise en charge interprofessionnelle: « Entre le fait que le médecin réfère à notre équipe douleur, puis que l’équipe le prend en charge. Moi, je vois un écart thérapeutique. » (P1).

Tel que présenté dans le Tableau 2, les gestionnaires identifient surtout l’évaluation interprofessionnelle (tous les projets), la collaboration interprofessionnelle (quatre des cinq projets) et la prise de décision partagée (trois des cinq projets) comme objectifs centraux de la mise en œuvre de ces innovations. La prise en charge interprofessionnelle de la douleur chronique, longtemps associée à des niveaux de services plus spécialisés, est ainsi repositionnée dans la mission des soins primaires, avec une prise de décision partagée et non centrée autour du médecin traitant :

C’est théoriquement une pathologie qui devrait être prise en charge en première ligne […] on est en train de vraiment les aider à développer leur offre de services et leur trajectoire [… Auparavant,] chez nous, la référence, c’était toujours un médecin qui voit le patient en premier et c’est le médecin qui va décider qui sont les professionnels […] mais là, la référence va se faire à une équipe dans laquelle il y a l’infirmière clinicienne […] qui va pouvoir, après une première rencontre, déterminer c’est quoi les bons professionnels. (P6)

Ce virage interprofessionnel s’accompagne d’une volonté d’améliorer la rapidité d’accès aux services « en donnant un accès plus rapide que les gens ont en deuxième ligne en ce moment. » (P3)

#### Objectif 2: Amélioration des mécanismes d’accès

La constitution de ces équipes a clairement permis d’améliorer le caractère continu du continuum de soins. Insérés entre les cliniques de soins primaires et de soins spécialisés, les projets réussissent tous à relier l’amont et l’aval du continuum bien que les partenariats avec les services spécialisés et les groupes de médecine familiale demeurent inégaux dans leurs formes (seulement deux des projets ont des réunions avec les GMF du territoire et la clinique spécialisée de la douleur conjointement). Les gestionnaires jugent que la communication avec les référents est modérément facile (médiane = 6,5/10 sur une échelle de 0 (fortement en désaccord) à 10 (fortement en accord)) et les services innovent localement : « On fait des partenariats avec les pharmacies communautaires […] documentation, courriels types remis [au patient] » (P7). Des efforts sont également déployés afin d’élargir la portée des services et de répondre au besoin de la population en dehors du continuum de douleur lui-même: « On a présenté notre projet à la table médicale territoriale […] les urgentologues peuvent nous référer des [patients]. » (P4). Ces actions témoignent d’une volonté de mieux arrimer les services et de réduire les discontinuités au sein du parcours de soins.

Les équipes appliquent des stratégies de référencement multiples et adaptatives, ciblant principalement les patients des GMF affiliés (tous les projets), les personnes en attente de services spécialisés (trois des cinq projets) et les patientes et les patients sans médecin de famille (trois des cinq projets). Les cas ne répondant pas aux critères définitoires d’une douleur chronique sont refusés par tous (parfois après une primo-évaluation), mais les cas les plus complexes sont cependant pris en charge de façon limitée (deux des cinq projets). La réduction des listes d’attente de services spécialisés constitue une priorité majeure : « Il y a 984 patients sur la liste d’attente de services spécialisés […] le médecin […] voulait revoir tous, tous, tous les dossiers […] pour décider quel patient va venir en première ligne. » (P3), surtout dans une perspective de démarrage des projets, ceux-ci privilégient les cas où les besoins sont les plus grands. La priorisation des patientes et des patients sans médecin de famille est considérée comme un enjeu d’ équité : « Eux, ils n’ont pas accès à un médecin de famille […] » (P7).

L’accès repose surtout sur un triage clinique interne systématique et centralisé par projet, avec des critères de priorisation variables selon les projets (p. ex., selon la durée de la douleur, le type de douleur, le niveau de complexité). Ces mécanismes, largement définis localement, manquent encore d’harmonization avec les services spécialisés en douleur. Parfois, ce sont les professionnelles et les professionnels de la santé des projets qui s’occupent de la priorisation des cas référés (deux des cinq projets), tandis que pour d’autres, ce sont les médecins référents (un des cinq projets), ou l’ordre d’arrivée de la requête (un des cinq projets) qui déterminent l’ordre dans lequel les patientes et les patients seront vus.

Malgré les différences dans l’organisation, notamment dans la composition des équipes, le rôle du médecin et les modalités d’accès et de priorisation, toutes les équipes partagent la même intention : améliorer le parcours des patientes et des patients dans la perspective de créer un continuum cohérent de services. Cette logique de continuité mène directement au deuxième axe du Plan d’action, centré sur le rôle actif de la patiente et du patient dans sa trajectoire de services.

### Axe 2 - Rôle de la patiente et du patient

Deux projets ont formellement identifié l’autonomisation des patientes et des patients comme l’un de leurs objectifs centraux d’implantation. En continuité avec l’approche centrée sur la personne, les équipes montrent un engagement envers l’autonomisation des patientes et des patients, même si les outils disponibles sont encore peu développés et peu uniformisés entre les projets (utilisation de projets web, développement de cahiers pour le participant et tenue d’ateliers thématiques). La patiente et le patient deviennent acteurs de leur parcours : *«* on va être beaucoup dans l’autoprise en charge, l’autogestion *»* (P6). L’objectif est de l’autonomiser dans la gestion à long terme de sa condition : « On leur apprend à vivre avec leurs douleurs (…) à vivre avec les deuils. » (P3). Cette orientation vers l’autogestion s’inscrit dans une vision d’accompagnement durable.

Les patientes et les patients partenaires sont des personnes ayant un vécu expérientiel de la douleur qui peuvent jouer un rôle actif soit au niveau de leurs soins (i.e., co-construit avec son équipe traitante des stratégies pour optimiser et adapter les traitements) ou soit au niveau de l’administration et la gestion des services (i.e., participe à la prise de décisions sur l’offre de services). Les patientes et les patients partenaires sont présents dans quatre des cinq projets, mais leurs rôles demeurent variables et surtout centrés sur les activités cliniques (trois des cinq projets). La participation aux décisions stratégiques ou à la planification clinique (un seul des cinq projets) reste encore ponctuelle et marginale. L’approche patient-partenaire est donc encore en développement, soutenue par la collaboration interprofessionnelle, mais peu formalisée.

### Axe 3 - Transfert des connaissances

Dans un contexte de création d’équipes et d’embauche de professionnelles et de professionnels de la santé de différents domaines, les gestionnaires rapportent un soutien clinique et expert élevé (médiane (écart interquartile): 8/10 (8–9)), notamment grâce aux formations continues disponibles, au programme ECHO (*Extension for Community Healthcare Outcomes*^21,22^) et au mentorat interprofessionnel. La majorité des professionnelles et des professionnels de la santé embauchés pour exécuter l’offre de service avait peu d’expérience dans le domaine de la douleur chronique, mais étaient très engagés: « Je tiens à mentionner que j’ai vraiment une super équipe, très impliquée, très dynamique » (P2). La formation des professionnelles et des professionnels en gestion de la douleur constitue donc un canal significatif de transfert des connaissances qui a été identifié par quatre des cinq projets et les gestionnaires insistent sur la nécessité d’un accès rapide et structuré à l’information : « S’assurer (de) la formation d’abord de ces équipes-là; s’assurer qu’il y ait un accès rapide à de l’information. » (P6). La formation des professionnelles et des professionnels à la collaboration interprofessionnelle n’a été identifiée comme un facilitateur que par deux des cinq projets. Les équipes des différents projets se supportent également d’une façon plus ou moins structurée : « On a eu quelques rencontres là, de deux à quatre par année, qui nous aident à échanger. On a demandé un canal TEAMS pour être capable de mieux communiquer avec eux. » (P2).

Ceci pose tout de même quelques enjeux relatifs à la création des offres de service dans un contexte où il y a initialement peu d’expertise à l’interne pour la gestion de la douleur chronique.

Pour implanter un projet comme ça … il faut savoir c’est quoi les meilleures pratiques en termes de douleur chronique … ok, on offre un service, mais est-ce que c’est la meilleure pratique? À mon avis, on n’a pas tous les outils. (P8)

Malgré cela, le transfert des connaissances sur la douleur chronique a une portée qui va au-delà des projets locaux pour rejoindre aussi les médecins en soins primaires:

Les médecins se sentaient pas du tout outillés pour gérer la douleur de leurs patients. Puis je parlais à un médecin la semaine passée, puis elle a dit j’ai l’impression, je fais juste donner des médicaments pour geler leur douleur, puis depuis que vous êtes là, je leur en donne moins, puis je me rends compte que ça avance quand même. (P3)

### Axe 4 - Évaluation et amélioration de la qualité

Des mécanismes de reddition de comptes et de suivi par le ministère et les Réseaux universitaires intégrés de santé et de services sociaux soutiennent la mise en œuvre des projets selon une approche personnalisée et adaptée aux réalités locales. De ça découle une volonté d’identifier les meilleures pratiques, dans la perspective d’une éventuelle mise à l’échelle à travers la province avec un projet dans chacun des centres intégrés de services de santé et de services sociaux et centres intégrés universitaires de santé et de services sociaux.

Malgré cette orientation stratégique et le soutien disponible, il y avait une grande hétérogénéité entre les projets au niveau des facteurs qui étaient perçus comme facilitant ou freinant la mise en place de conditions organisationnelles pour améliorer la qualité de la pratique. Par exemple, la coordination de l’équipe avec les autres partenaires était perçue comme un facilitant par un des cinq projets et comme un frein par un autre des projets. La coordination interne (facilitateur : deux des cinq projets; frein : un des cinq projets), les processus de définition des critères d’accès et de priorisation des services (facilitateur : deux des cinq projets), et le développement d’outils cliniques (facilitateur : deux des cinq projets; frein : deux des cinq projets) pouvaient être à la fois des conditions facilitantes ou des freins en fonction des projets. Les gestionnaires soulignent la lourdeur administrative (trois des cinq projets) dont la collecte de données et des enjeux organisationnels récurrents : On doit « recueillir des données, puis on comprend, c’est tout à fait normal, mais le tableau (est) interminable, très difficile à comprendre […]. Donc il a vraiment fallu débroussailler. » (P5). Malgré tout, l’incertitude a généré une forte culture adaptative des gestionnaires qui, par exemple, convergent peu à peu vers les meilleures pratiques (p. ex., articulation des offres de service qui propose un équilibre entre les interventions individuelles et de groupe).

### Axe 5 - Gouvernance

Des rencontres régulières sont organisées entre le ministère et les gestionnaires de chacun des projets individuellement afin de suivre l’évolution des projets, de soutenir les équipes, de favoriser la reddition de compte et dans une moindre proportion de créer une opportunité de partage entre les équipes. Le ministère soutient les équipes en apportant des pistes de solutions aux défis rencontrés, utilise son positionnement stratégique pour faciliter le réseautage et l’intégration des cliniques dans le continuum de soins, stimule les équipes vers l’atteinte des cibles, opérationnalise les processus au travers du continuum de soins et facilite la gestion de changement dans le réseau pour une meilleure intégration des projets.

On a de bonnes relations avec nos partenaires au ministère. Je me sens quand même soutenue par le ministère. Si j’ai des questions ou quoi que ce soit, un courriel, ils nous répondent, une petite rencontre de quinze minutes. Je pense que du côté du ministère, ma supérieure immédiate puis son agente de planification, de programmation et de recherche, c’est pas mal des bons piliers là dans l’élaboration de ce projet-là. Elle contribue à tout ce qui est indicateur, Plan d’action, reddition de comptes. Elle va nous aider à un peu structurer. (P4)

D’ailleurs, plusieurs facilitateurs à la gestion des services ont été identifiés par les gestionnaires, notamment l’explicitation d’une stratégie de changement (trois des cinq projets) et une définition des objectifs des services offerts (trois des cinq projets). L’incertitude financière se fait par contre sentir: « On n’a pas la garantie que ça va être pérennisé … on a le financement sur deux ans, on n’a pas l’information pour la suite. » (P4) Ces contraintes se doublent de problèmes d’espaces et d’infrastructures: « Il y a des locaux qui avaient été ciblés pour faire le projet […] qui ont été récupérés par d’autres » (P6). Le roulement de personnel contribue aussi à fragiliser la continuité des services et de la prise en charge des patientes et des patients: « J’ai eu un roulement incroyable […] il restait juste moi du projet initial. » (P2) Les gestionnaires identifiaient également le besoin de faire des compromis entre ce qui serait optimal et réalistiquement ce qui peut être fait selon les balises entourant la mise en place de ces services:

(Interviewer) Avez-vous sélectionné vos employés sur la base du fait qu’ils étaient déjà très compétents et très engagés en douleur chronique ?

(P3): Pas du tout … On n’avait pas le choix de toute façon, parce qu’on affiche des postes, c’était des surcroîts qu’on a affichés, c’était les personnes intéressées qui sont venues vers nous.

Des disparités existent également dans l’interprétation du mandat et des attentes, ce qui découle en partie du processus de maturation du dispositif d’accompagnement et de la variété des cadences de développement des initiatives locales. Les innovations mises en place diffèrent principalement au niveau des sources de référencement, des types de professions représentées dans les membres de l’équipe clinique, le type d’accompagnement et de soins offert aux patients, ainsi que les critères d’éligibilité et durée de l’accompagnement. Bien que ceci offre l’avantage de pouvoir mieux comprendre le type de modèle qui fonctionne le mieux, plusieurs gestionnaires auraient souhaité des directives plus précises : « J’aurais voulu avoir peut-être un meilleur descriptif de la mission » (P8), ainsi qu’un accompagnement dans la mise en œuvre adapté à ces réalités locales : « Je pense que le ministère pourrait nous soutenir de manière peut-être un petit peu plus étroite […] pour savoir comment, c’est quoi les enjeux qu’on vit avec l’implantation des programmes » (P8). Certains freins à la gestion des services ont été observés, notamment l’opérationnalisation des ressources (trois des cinq projets), la charge de travail additionnelle (trois des cinq projets), et les échéanciers très courts pour déployer les services (deux des cinq projets). La difficulté de recrutement des cliniciennes et des cliniciens était également identifiée comme influençant grandement les conditions de gestion des services, notamment pour les physiothérapeutes (trois des cinq projets) et les psychologues (deux des cinq projets).

## Discussion

L’implantation d’équipes interprofessionnelles en soins primaires dédiées à la douleur chronique constitue un tournant majeur dans l’organisation des soins au Québec. Ces projets misent sur l’accessibilité, le soutien à l’autonomisation et une prise en charge interprofessionnelle mieux adaptée aux besoins réels de la population. Ils introduisent, en soins primaires, des pratiques interprofessionnelles auparavant peu présentes pour la prise en charge de la douleur chronique dans le contexte québécois et renforcent la continuité des soins en reliant les soins primaires et spécialisés. Ils ajoutent une capacité dédiée au renforcement de l’autogestion de la douleur chronique comprise et appréciée par les patientes et les patients.

La littérature scientifique démontre que les programmes interprofessionnels de soins primaires améliorent l’autogestion, le fonctionnement quotidien et une prise en charge multidimensionnelle.^12^ Contrairement au modèle ancré en soins spécialisés qui fragmente les soins,^23^ les équipes interprofessionnelles en première ligne permettent une réponse simultanée aux différentes dimensions de la douleur, ce qui confirme la pertinence du modèle québécois et l’importance d’en assurer la pérennité. Toutefois, la mise en œuvre concrète demeure difficile^24^ et dépend tant des ressources disponibles que du cadre organisationnel. Cette phase d’expérimentation permet néanmoins d’identifier les pratiques les mieux adaptées au contexte québécois.

Ces résultats sont cohérents avec l’organization de services interprofessionnels en soins primaires pour les personnes présentant une multimorbidité.^25^ L’évaluation de l’implantation de ces services identifie plusieurs éléments des caractéristiques de l’intervention (qualité et complexité de l’intervention), du contexte externe (besoins et ressources, politiques externes), du contexte interne (caractéristiques structurelles, réseautage, communication, leadership, culture), des caractéristiques des gestionnaires (connaissances et croyances), et des processus (planification, évaluation, présence de champions) qui influencent la réussite de leur mise en œuvre.

### Difficultés d’opérationnalisation malgré un large consensus international

Les recommandations nationales et internationales insistent clairement sur la nécessité de déployer de tels modèles.^26–28^ Pourtant, comme le montrent Piano et al.,^29^ même dans des contextes dotés de plans nationaux robustes, la mise en œuvre reste fragmentée et marquée par des disparités, un manque de coordination et un épuisement des ressources humaines.^29^ Ces limites rejoignent celles décrites par Seal et al.^11^ qui montrent que de nombreux systèmes demeurent ancrés dans un modèle biomédical favorisant les interventions techniques et les traitements pharmacologiques.^5^ Cette logique de silos limite la collaboration interprofessionnelle, restreint le partage d’information et empêche l’élaboration d’une vision commune de la patiente et du patient, entravant ainsi l’opérationnalisation d’un modèle réellement interprofessionnel.^23^ Les résultats de la présente étude révèlent des enjeux comparables au chapitre de l’implication médicale périphérique et du développement de la coordination à travers le continuum de soins. Ainsi, même si les principes biopsychosociaux sont reconnus, leur mise en pratique se heurte à des contraintes organisationnelles profondes. Pourtant, quand la collaboration interprofessionnelle et le travail en équipe sont déployés au sein de programmes interprofessionnels en soins primaires, il y a une amélioration de la condition douloureuse des personnes prises en charge par ces services.^6^

### Beaucoup de besoins, peu de ressources

La prévalence élevée de la douleur chronique, la capacité restreinte des services spécialisés et la pression mise sur les soins primaires sont des défis majeurs ici comme ailleurs.^29,30^ Seule une faible proportion de personnes accède aux services spécialisés, ce qui exerce une forte pression sur les soins primaires. Or, les médecins en soins primaires ne peuvent appliquer seuls les lignes directrices en douleur chronique, qui exigent un soutien interprofessionnel continu.^11^ Dans la mise en place initiale des projets, les médecins ont été initialement peu impliqués, ce qui reflète une coordination encore inégale et une capacité variable à s’intégrer dans des équipes interprofessionnelles. La mise en oeuvre s’est ainsi déployée davantage en fonction des ressources disponibles que par le modèle souhaité. Le rôle des infirmières et des infirmiers dans les projets semble central et permet de mettre de l’avant une pratique prometteuse en douleur chronique en soins primaires. En effet, le personnel infirmier possède une expertise en gestion des maladies chroniques déjà démontrée et qui pourrait être davantage utilisée en douleur chronique, malgré la présence de plusieurs défis.^31^

Plusieurs des services ont identifiés des défis de recrutement et de rétention des différents professionnelles et professionnels de la santé et ceux-ci étaient souvent peu formés pour œuvrer spécifiquement auprès de la patientèle en douleur chronique. Ceci semble être lié à l’organisation du système de santé et les processus de dotation, ainsi qu’au fait que les postes affichés étaient des postes temporaires. Malgré tout, les équipes rapportaient un engagement élevé des professionnelles et professionnels embauchés envers la mission de la douleur chronique, mais réitère en même temps l’importance d’avoir accès rapidement à de la formation sur la gestion de la douleur chronique et l’accès à des ressources et du soutien. L’*International Association for the Study of Pain* a d’ailleurs rédigé plusieurs curriculums sur les connaissances requises pour la gestion de la douleur chronique pour différents types de professionnels de la santé^32^ et qui pourrait guider les besoins de formation des équipes sur le terrain.

Les défis associés à une prise en charge interprofessionnelle sont cohérents avec ceux révélés par Brooks et collègues aux États-Unis, notamment un besoin de connaissances et de confort accrus pour gérer la douleur chronique auprès des médecins, des enjeux de rémunération, ainsi que le peu de ressources disponibles.^33^ Un scan environnemental sur les modèles de soins en douleur chronique au Canada et ailleurs révèle également que les barrières les plus fréquemment rapportées incluent le financement, le soutien, et la collaboration avec le gouvernement et les instances locales.^34^

### Un cadre organisationnel qui limite la cohérence et la pérennité du modèle

La présente étude relève également plusieurs freins organisationnels qui limitent la cohérence et la pérennité du modèle. La lourdeur administrative, le financement temporaire, les défis associés à la gestion du changement et l’instabilité des ressources humaines compromettent l’intégration harmonieuse des différentes dimensions du modèle et la continuité des pratiques interprofessionnelles. Ces obstacles relèvent moins d’une faiblesse du modèle que des contraintes structurelles dans lesquelles il se déploie et du stade préliminaire de ces projets pilotes.

### Forces et limites de l’étude

L’utilisation d’un devis multiméthode permet d’obtenir un portrait plus complet de l’implantation des projets. Toutefois, le nombre restreint de projets examinés (cinq sur les six déployés) qui se situaient exclusivement au Québec et la petite taille de l’échantillon pour les données quantitatives limitent la portée et la transférabilité des résultats. De plus, le contexte organisationnel en évolution constante au moment de la collecte des données pourrait avoir influencé certaines réponses.

## Conclusion

Cette étude montre que l’implantation de projets interprofessionnels en soins primaires pour la douleur chronique est possible et constitue une avancée importante au Québec, mais la situation demeure fragile. Les équipes ont réussi à intégrer des pratiques biopsychosociales et à améliorer l’accessibilité et la coordination locale des soins, mais ont constaté des défis persistants liés au manque de ressources et aux contraintes organisationnelles. Pour assurer la pérennité d’un tel type de modèle, un soutien institutionnel accru, une harmonisation des pratiques et un financement stable apparaissent essentiels.

## Supplementary Material

EVADO_Annexe 1.pdf

coreq.pdf

## Financement

Ces travaux ont été supportés par une subvention des Instituts de recherche en santé du Canada (Institut de l’appareil locomoteur et de l’arthrite et la Stratégie de recherche axée sur le patient) conjointement avec les [no 202211IT6]. MG Pagé est supportée par une bourse salariale Junior 2 des Fonds de recherche du Québec en santé.
