# Supplementary material for: Processus organisationnels et pratiques cliniques pour la gestion de la douleur chronique en soins primaires : une offre de service découlant du plan d’action en douleur chronique 2021-2026 ministériel du Québec, Canada
Source: Can J Pain. 2026 Jul 9;10(1):2672488. doi: 10.1080/24740527.2026.2672488 (PMC13353775; doi:10.1080/24740527.2026.2672488)
Supplement: EVADO_Annexe 1.pdf [file UCJP_A_2672488_SM3489.pdf]

Questionnaire d'enquête organisationnelle
Ce questionnaire s'adresse à
la personne responsable de la gestion de l'équipe interprofessionnelle

Dans le contexte de la mise en œuvre du Plan d'action ministériel en douleur chronique et de l'implantation des équipes interprofessionnelles, ce questionnaire a pour but de brosser un portrait de l'évolution du continuum des soins et services en douleur chronique. Le même questionnaire sera distribué une 2e fois dans 2 ans et une 3e fois dans 4 ans dans le but d'évaluer des changements au fil du temps. Il n'y a pas de bonnes ou mauvaises réponses. Répondez au meilleur de votre connaissance.
Ici, l'expression > fera référence aux professionnels de la santé non-médecins.

Vous répondez à ce questionnaire en tant que :

◯ Professionnel de l'équipe interprofessionnelle et responsable de la gestion de l'équipe
◯ Gestionnaire du CI(U)SSS responsable de l'équipe interprofessionnelle
◯ Autre ______
◯ Je préfère ne pas répondre

IMPLANTATION DE L'ÉQUIPE INTERPROFESSIONNELLE

L'expression > fait référence ici à l'équipe de professionnels mise sur pied en services de proximité pour les personnes qui vivent avec de la douleur chronique et qui ont des besoins complexes nécessitant l'intervention de plusieurs professionnels spécialisés (par ex., psychologues, infirmières, pharmaciens, etc.).

1. Dans le cadre de l'implantation de l'équipe interprofessionnelle en douleur chronique, cochez l'affirmation qui correspond le mieux à votre perception de la latitude qu'on vous laisse pour adapter vos pratiques aux besoins et aux soins des personnes qui présentent de la douleur chronique.

◯ On nous laisse trop de latitude
◯ On nous laisse toute la latitude nécessaire
◯ On nous laisse suffisamment de latitude
◯ On ne nous laisse pas assez de latitude
◯ On ne nous laisse aucune latitude
◯ Je préfère ne pas répondre

2. Parmi les objectifs spécifiques énoncés dans le Plan d'action ministériel en douleur chronique, identifiez les objectifs qui ont guidé prioritairement le développement de votre projet de formation d'une équipe interprofessionnelle (vous pouvez en identifier plus d'un, mais ne sélectionnez que ceux qui sont prioritaires)

☐ Favoriser une évaluation globale et multidisciplinaire du patient afin de mieux répondre à ses besoins
☐ Favoriser l'autonomie des patients
☐ Favoriser la prise de décision partagée entre le patient et le professionnel de la santé
☐ Favoriser la collaboration interprofessionnelle
☐ Mettre en place la fonction gestion de cas
☐ Soutenir la formation des professionnels en douleur chronique
☐ Contribuer à l'amélioration de la collaboration entre les différents niveaux de soins
☐ Améliorer les trajectoires d'accès au plateau diagnostique en douleur chronique
☐ Améliorer les trajectoires d'accès aux interventions techniques
☐ Assurer une représentation des patients partenaires au sein de l'équipe
☐ Nous avons ajouté d'autres objectifs à ceux-ci.
☐ Les besoins de notre population ne correspondaient pas tout à fait à ces objectifs.
☐ Nous avons plutôt développé nos propres objectifs.
☐ Nous n'avions pas d'objectifs spécifiques
☐ Je préfère ne pas répondre

2.1. En quelques lignes, énoncez les objectifs plus spécifiques à votre équipe interprofessionnelle ?

3. Identifiez la ou les populations ciblées en priorité dans le développement de votre projet (maximum de 3)

- ☐ Offrir des services en priorité pour les patients ayant des besoins complexes
- ☐ Offrir des services en priorité aux cas les plus urgents
- ☐ Offrir des services en priorité aux personnes qui sont sur la liste d'attente de cliniques spécialisées en douleur chronique
- ☐ Offrir des services en priorité aux personnes desservies par les GMF(u) affiliés avec votre CI(U)SSS
- ☐ Offrir des services en priorité aux personnes qui n'ont pas de médecin de famille et qui vivent avec de la douleur chronique
- ☐ Offrir des services à un plus grand nombre de personnes qui vivent avec de la douleur chronique
- ☐ Autre ______
- ☐ Je préfère ne pas répondre

4. Y a-t-il des patients partenaires/experts dans votre projet d'implantation de l'équipe interprofessionnelle ?

- ◯ Oui
- ◯ Non, ce n'était pas dans nos plans
- ◯ Non, nous n'avons pas réussi à recruter un patient partenaire bien que ce fût notre intention
- ◯ Je préfère ne pas répondre

4.1. Comment se traduit la participation des patients partenaires/experts à l'implantation de votre projet ?

- ☐ Ils ont contribué à définir les objectifs spécifiques
- ☐ Ils contribuent à identifier les besoins des personnes qui vivent avec de la douleur chronique
- ☐ Ils participent à nos réunions de travail
- ☐ Ils s'impliquent auprès de patients qui ont des besoins complexes
- ☐ Ils participent aux activités que nous organisons pour les patients
- ☐ Aucune de ses affirmations
- ☐ Autre contribution ______
- ☐ Je préfère ne pas répondre

5. Parmi les ressources que vous souhaitiez recruter dans votre équipe, cochez les services pour lesquels il vous a été impossible de recruter.

- ☐ Personnel en réadaptation - physiothérapeute
- ☐ Personnel en réadaptation - kinésiologue
- ☐ Personnel en réadaptation - autre ______
- ☐ Personnel en psychosocial - psychologue
- ☐ Personnel en psychosocial - travailleur social
- ☐ Personnel en psychosocial - autre ______
- ☐ Personnel en pharmacie
- ☐ Personnel en médecine (médecin responsable)
- ☐ Personnel en nutrition
- ☐ Personnel autre ______
- ☐ Le recrutement de personnel n'a pas été un enjeu important
- ☐ Je préfère ne pas répondre

5.1. Si le recrutement a été un enjeu, comment expliquez cette difficulté, selon vous?
(Expliquez brièvement, SVP)

6. Identifiez les secteurs d'activité qui ont été les plus difficiles à gérer dans l'implantation de votre projet (vous pouvez sélectionner plus d'un choix de réponse).

☐ Définir une stratégie explicite de gestion du changement
☐ Définir les objectifs du projet
☐ Précisez l'ampleur du projet
☐ Gérer un projet avec un échéancier très court
☐ Gérer un projet à durée possiblement limitée
☐ Opérationnaliser le projet; p. ex, la disponibilité et l'aménagement des espaces
☐ Coordonner l'équipe avec les autres partenaires (Ex. : GMF, clinique spécialisée, MSSS, équipe de recherche)
☐ Coordonner l'équipe elle-même
☐ Définir le modèle d'accessibilité/priorisation
☐ Développer les outils
☐ Assurer la formation des professionnels en gestion de la douleur
☐ Assurer la formation des professionnels à la collaboration interprofessionnelle
☐ Assumer la charge de travail venant s'ajouter aux responsabilités déjà en cours
☐ Gérer la collecte des données : indicateurs prévus par le MSSS
☐ Autre ______
☐ Je préfère ne pas répondre

7. Parmi ces mêmes secteurs d'activité, identifiez ceux qui vous ont été faciles à gérer dans l'implantation de votre projet.

☐ Définir une stratégie explicite de gestion du changement
☐ Définir les objectifs du projet
☐ Précisez l'ampleur du projet
☐ Gérer un projet avec un échéancier très court
☐ Gérer un projet à durée possiblement limitée
☐ Opérationnaliser le projet; p. ex, la disponibilité et l'aménagement des espaces
☐ Coordonner l'équipe avec les autres partenaires (GMF, clinique spécialisée, MSSS, équipe de recherche)
☐ Coordonner l'équipe elle-même
☐ Définir le modèle d'accessibilité/priorisation
☐ Développer les outils
☐ Assurer la formation des professionnels en douleur chronique
☐ Assurer la formation des professionnels à la collaboration interprofessionnelle
☐ Assume la charge de travail venant s'ajouter aux responsabilités déjà en cours
☐ Gérer la collecte des données : indicateurs prévus par le MSSS
☐ Autre ______
☐ Je préfère ne pas répondre

8. Y a-t-il eu des éléments qui ont facilité l'implantation de votre projet

PRISE EN CHARGE DES PATIENTS/AUTOGESTION

1. Est-ce que vous disposez d'outils pour guider la prise en charge des patients qui vivent avec de la douleur chronique?

☐ Protocoles de soins interprofessionnels
☐ Ordonnance collective
☐ Outils d'aide à la décision
☐ Communauté de pratique
☐ Autre ______
☐ En développement ______
☐ Aucun
☐ Je préfère ne pas répondre

2. Dans votre équipe, utilisez-vous un plan de soins (démarche impliquant une évaluation des besoins permettant de structurer la dispensation des interventions) pour les patients qui vivent avec de la douleur chronique?

◯ Oui, nous utilisons formellement un plan de soins pour chacun de ces patients
◯ Nous n'avons pas développé cet outil
◯ Oui, mais pour certains patients seulement
◯ Je préfère ne pas répondre

2.1. Cochez ce qui s'applique le mieux à votre démarche. (un seul choix)

◯ Le plan de soins est élaboré en collaboration avec le patient
◯ Le plan de soins est élaboré en collaboration avec le patient et ses proches
◯ Le patient est peu impliqué dans l'élaboration du plan de soins
◯ Je préfère ne pas répondre

2.1. SVP précisez pour quels patients spécifiques vous utilisez un plan de soins.

2.2. Comment est rédigé le plan de soins ?

◯ Chaque professionnel de l'équipe évalue les besoins du patient selon son domaine de compétence et rédige son plan de soins
◯ Un seul des professionnels de l'équipe évalue les besoins du patient et rédige le plan de soins
◯ Un seul professionnel de l'équipe évalue les besoins du patient et propose un plan de soins à l'équipe
◯ Autre ______
◯ Je préfère ne pas répondre

2.3. Est-ce qu'un médecin contribue à l'élaboration du plan de soins?

◯ Oui, celui-ci contribue à l'élaboration du plan de soins
◯ Oui, celui-ci approuve le plan de soins élaboré par un professionnel
◯ Non
◯ Autre approche ______
◯ Je préfère ne pas répondre

---

3. À quelle fréquence les visites de suivi sont-elles prévues pour les patients qui vivent avec de la douleur chronique.

◯ 1 fois/2 semaines
◯ 1 fois/mois
◯ 1 fois/2 mois
◯ 1 fois/3 mois
◯ 1 fois/6 mois
◯ 1 fois/12 mois
◯ Autres ______
◯ Les suivis se font au besoin seulement
◯ Je préfère ne pas répondre

---

4. Prise en charge des patients avec des besoins complexes. Cochez ce qui correspond le mieux à votre équipe.

◯ Les patients ayant des besoins complexes sont identifiés formellement et notre équipe a développé un plan de soins spécifique pour eux
◯ Les patients ayant des besoins complexes sont référés à la clinique spécialisée en douleur chronique
◯ Les patients avec des besoins complexes ne sont pas identifiés formellement
◯ Nous n'avons pas d'outils pour identifier les patients avec des besoins complexes
◯ Je préfère ne pas répondre

5. À quelles ressources d'éducation ont accès vos patients pour leur expliquer en quoi consiste la douleur chronique ? Plus d'une réponse possible.

☐ Notre équipe interprofessionnelle a développé des outils spécifiques
☐ L'éducation est faite par le professionnel qui suit ces patients
☐ La clinique spécialisée en douleur chronique de notre CI(U)SSS offre des formations pour aider les personnes qui vivent avec de la douleur chronique
☐ Notre équipe favorise l'accès à la plateforme Gérer ma douleur
☐ Notre équipe favorise les ressources de l'Association québécoise de la douleur chronique
☐ Autre ______
☐ Je préfère ne pas répondre

FONCTIONNEMENT DE L'ÉQUIPE INTERPROFESSIONNELLE

1. Y a-t-il un médecin appartenant à un GMF(U) qui est spécifiquement associé à votre équipe interprofessionnelle pour les patients vivant avec de la douleur chronique ?

◯ Oui
◯ Non, associer un médecin à notre équipe n'était pas un objectif
◯ Non; il y a un besoin mais nous n'avons pas réussi à identifier cette personne
◯ Je préfère ne pas répondre

2. À quelle fréquence les professionnels de votre équipe ont-ils des réunions formelles pour discuter et coordonner les soins des patients qui vivent avec de la douleur chronique.

◯ 1 fois/semaine
◯ 1 fois/2 semaines
◯ 1 fois/mois
◯ 1 fois /2 mois
◯ Les professionnels de notre équipe ont plutôt des discussions informelles pour coordonner les soins aux patients qui vivent avec de la douleur chronique
◯ Aucune de ces réponses
◯ Je préfère ne pas répondre

ACCESSIBILITÉ À VOTRE ÉQUIPE ET PRIORISATION

1. Quelle est la procédure d'accès à votre équipe ? Cochez tout ce qui s'applique.

☐ Le médecin de famille du GMF(U) ou un professionnel du GMF(U) fait une demande de service à un professionnel spécifique (infirmière, pharmacien, physiothérapeute, etc.) de votre équipe
☐ Le médecin de famille du GMF(U) ou un professionnel du GMF(U) fait une demande de service à votre équipe pour un problème de douleur chronique et vous laisse le soin d'évaluer quel professionnel de votre équipe répondra le mieux aux besoins du patient
☐ Nous identifions les patients à partir de la liste d'attente de la clinique spécialisée en douleur chronique
☐ Nous accueillons les patients à partir du guichet d'accès à la 1re ligne
☐ Notre procédure est en développement
☐ Si aucune de ces options, : ______
☐ Je préfère ne pas répondre

2. Comment est priorisé l'accès à vos services ?

◯ Le professionnel ou le médecin du GMF(U) qui a fait la demande indique lui-même le niveau de priorisation
◯ Un professionnel de notre équipe consulte le DME et communique avec le patient par téléphone pour une première évaluation et priorise ensuite
◯ Les rendez-vous sont donnés par ordre d'arrivée
◯ Autre processus de priorisation ______
◯ Aucun critère de priorisation n'a été défini
◯ Je préfère ne pas répondre

3. Comment votre équipe a-t-elle déterminé les critères d'accessibilité et de priorisation ?

◯ Notre équipe a elle-même déterminé ses critères de priorisation
◯ Note équipe a déterminé des critères de priorisation avec l'équipe GMF(U)
◯ Notre équipe a déterminé des critères de priorisation avec la clinique spécialisée de notre CI(U)SSS
◯ Notre équipe a déterminé des critères de priorisation avec l'équipe GMF(U) et la clinique spécialisée de notre CI(U)SSS
◯ Autre ______
◯ Je préfère ne pas répondre

1. Les intervenants de la clinique spécialisée en douleur chronique de votre CI(U)SSS sont disponibles pour offrir du soutien (p ex. des avis d'experts)

______
Fortement

en désaccord

Fortement

en accord

2. Lorsqu'un patient est référé à votre équipe par un médecin ou un professionnel du GMF(U), il est facile de communiquer avec ceux-ci pour discuter des besoins du patient.

______
Fortement

en désaccord

Fortement

en accord

SVP, sélectionner "Non applicable" lorsque vous ne communiquez pas avec le médecin/professionnel référant.

3. Coordination et harmonisation des services. Cochez ce qui correspond le mieux à votre équipe.

◯ Votre équipe a des réunions réunissant à la fois l'équipe de la clinique spécialisée et le ou les GMF(U) pour discuter de la coordination des soins des patients qui vivent avec de la douleur chronique; spécifiez la fréquence. ______
◯ Votre équipe a des réunions formelles uniquement avec la clinique spécialisée en gestion de la douleur chronique; spécifiez la fréquence. ______
◯ Votre équipe a des réunions uniquement avec les équipes GMF(U) impliquées dans le projet d'implantation; spécifiez la fréquence. ______
◯ Aucune de ces options.
◯ Je préfère ne pas répondre

STRUCTURE DE VOTRE ÉQUIPE

1. Identifiez les professionnels qui composent votre équipe et spécifiez le nombre d'heures par semaine qu'ils consacrent à la douleur chronique.

☐ Ergothérapeute ______
☐ Infirmière ______
☐ Infirmière clinicienne ______
☐ Infirmière praticienne spécialisée ______
☐ Kinésiologue ______
☐ Médecin de famille ______
☐ Nutritionniste ______
☐ Physiothérapeute ______
☐ Pharmacien ______
☐ Psychologue ______
☐ Travailleur social ______
☐ Gestionnaire ______
☐ Adjointe administrative ______
☐ Autre ______
☐ Je peréfère ne pas répondre

____

2. À quel endroit se situe votre équipe interdisciplinaire ?

☐ Dans un GMF
☐ Dans un CLSC
☐ Dans un hôpital
☐ Dans un autre endroit ______
☐ Je préfère ne pas répondre

3. Avez-vous un système de gestion de données vous permettant de connaître le nombre de patients avec douleur chronique qui sont suivis dans votre clinique ?

◯ Oui
◯ Non
◯ Je préfère ne pas répondre

3.1. Quel est le nombre de patients qui vivent avec de la douleur chronique et qui ont été admis dans votre équipe depuis vos débuts comme équipe?

____

3.1. Quel est le nombre approximatif de patients qui vivent avec de la douleur chronique et qui ont été admis dans votre équipe depuis vos débuts comme équipe?

____

Si vous avez des commentaires ou des points que vous souhaitez ajouter, veuillez le faire ici.

MERCI DE VOTRE PRÉCIEUSE COLLABORATION
